# A Practical Cardiovascular Health Assessment for Manual Wheelchair Users During the 6-Minute Push Test †

**DOI:** 10.3390/s25072313

**Published:** 2025-04-05

**Authors:** Maja Goršič, Madisyn R. Adelman, Grace McClatchey, Jacob R. Rammer

**Affiliations:** 1 Department of Biomedical Engineering, University of Wisconsin-Milwaukee, Milwaukee, WI 53211, USA; adelman3@uwm.edu (M.R.A.); gofasipe@uwm.edu (G.M.); jrrammer@uwm.edu (J.R.R.); 2 Department of Biomedical Engineering, Marquette University, Milwaukee, WI 53233, USA

**Keywords:** cardiovascular health, cardiovascular assessment, manual wheelchair users, oxygen consumption, heart rate, VO_2max_, physical exertion, 6-Minute Push Test

## Abstract

Traditional VO_2max_ testing methods are often impractical for manual wheelchair users, as they rely on lower-body exercise protocols, require specialized equipment, and trained personnel. The 6-Minute Push Test (6MPT) is a widely used cardiovascular assessment that may provide a feasible alternative for estimating aerobic capacity in this population. This study aimed to develop a predictive model for VO_2max_ using physiological variables recorded during the 6MPT. Twenty-eight participants (14 novice and 14 expert manual wheelchair users) completed the test while wearing a VO_2_ mask and heart rate monitor. Spearman correlation analysis showed that distance covered during the 6MPT significantly correlated with VO_2max_ (r = 0.685, *p* < 0.001). A stepwise linear regression identified two predictive models: one using distance alone (R^2^ = 0.416, *p* < 0.001) and another incorporating both distance and maximum heart rate (R^2^ = 0.561, *p* < 0.001). These models offer practical estimations of VO_2max_, eliminating separate protocols. Our findings suggest that the 6MPT can serve as a simple, cost-effective alternative to laboratory-based VO_2_ testing, facilitating routine cardiovascular fitness assessments for manual wheelchair users in clinical and community settings. Future research should focus on validating these models in a larger, more diverse cohort to enhance their generalizability.

## 1. Introduction

Manual wheelchair use is a primary means of mobility for millions in the United States and many more around the world [1]. However, manual wheelchair users often lead a more sedentary lifestyle [2], which can hinder their mobility and functional independence [3,4]. Prolonged inactivity may also contribute to secondary health conditions, including pressure sores, musculoskeletal problems, and cardiovascular disease. Therefore, regular movement and physical activity are vital for maintaining the health and well-being of manual wheelchair users to help prevent these secondary conditions [4,5,6,7,8], emphasizing the importance of assessing both fitness and cardiovascular health in this population.

Cardiovascular fitness plays a crucial role in the overall health and quality of life for manual wheelchair users [3,5]. One means of assessing their health and fitness is through the maximal rate of oxygen consumption (VO_2max_), a key indicator of aerobic capacity and overall cardiovascular health. VO_2max_ measurements can provide valuable insight into the physical demands of those with mobility impairments [9,10,11,12].

Traditional VO_2max_ testing methods involve high-intensity physical activities, such as running on a treadmill or cycling on an ergometer [13]. Several studies have demonstrated that specific physiological responses and performance metrics, such as heart rate, speed, and distance, measured during tests, correlate with VO_2_ consumption. Heart rate, in particular, has been shown to closely align with VO_2_ levels [11], even after high-intensity training [14,15]. Additionally, improvements in cardiovascular fitness have been associated with increases in VO_2max_ [12,15]. As an alternative to treadmill testing, the 6-Minute Run Test has been used to measure VO_2max_ [16]. Similarly, the distance covered during the 6-Minute Walk Test has previously been used to predict VO_2max_ in healthy individuals [17,18], suggesting that a comparable relationship may exist for manual wheelchair users.

For manual wheelchair users, the 6-Minute Push Test (6MPT), adapted from the 6-Minute Walk Test [19], is a commonly used assessment of physical and cardiovascular health. The 6MPT measures the distance a manual wheelchair user can propel themselves in 6 min [20]. The test serves as a practical and accessible tool, providing insights into cardiovascular health and overall fitness levels [4,9,21,22,23,24], essential for daily mobility and independence. Studies on 6MPT have conducted VO₂ measurements separately from the test, requiring two distinct testing protocols (VO_2_ test and 6MPT) adding time and logistical burden [9,21,22].

Typical VO_2max_ testing protocols are not designed for upper body propulsion, making them especially challenging for manual wheelchair users. Additionally, these tests may require specialized laboratory equipment and trained personnel, further limiting their accessibility. While these traditional methods are highly accurate and reliable [13,21,25], they are often expensive, require an extensive setup, and add complexity to the testing process. As a result, they are often impractical for regular cardiovascular assessment in community settings [26]. These limitations underscore the need for a simpler, field-based method to estimate VO_2max_, specifically tailored to the functional abilities of manual wheelchair users.

To our knowledge, despite the apparent correlation that exists between physiological responses and VO_2_ consumption, the specific relationship and predictive formulas for manual wheelchair users have not yet been reported, apart from our previous work with limited data [27]. The 6MPT can provide measurable indicators of cardiovascular health and fitness in this population, yet its potential for VO_2max_ estimation remains unexamined. We hypothesized that the performance and physiological metrics measured during the 6MPT would be a significant predictor of VO_2max_ and could be used to develop a practical estimation model for manual wheelchair users. The goal of this study is to develop a simple formula for VO_2_ estimation that could offer a practical, cost-effective alternative to direct VO_2_ testing, thus providing a valuable tool for routine cardiovascular fitness assessment for manual wheelchair users beyond clinical settings.

## 2. Materials and Methods

This study was reviewed and approved by the University of Wisconsin—Milwaukee Institutional Review Board (IRB#23.347).

### 2.1. Participants

Twenty-eight participants were selected for this study: 14 expert manual wheelchair users and 14 novice manual wheelchair users. A participant was considered an expert manual wheelchair user if they utilized a manual wheelchair for everyday mobility or intense physical activity, and a participant was considered a novice manual wheelchair user if they had never used a manual wheelchair before the study. Participants were selected based on the following inclusion criteria: at least 18 years of age, physically capable of propelling themselves in a manual wheelchair, no record of epilepsy, claustrophobia or increased light sensitivity, and free from any upper extremity injury or surgery within the past year. Additionally, participants with health issues or taking medications relating to blood pressure, heart, or cardiovascular conditions were directed to consult their provider before participating.

Once informed consent was obtained, each participant started their lab appointment with a demographic questionnaire, which reported their age, height, gender, weight, injury level (if applicable), and self-reported weekly activity level. The participants used weekly activity measures categorized as high (more than 3 h/week), moderate (2–3 h/week), or low (under 2 h/week) [28,29].

The demographic characteristics of the participants are summarized in Table 1. Among the 14 novice wheelchair users, 8 reported a high level of weekly activity, 4 reported a moderate level, and 2 reported a low level. In contrast, of the 14 long-term wheelchair users, 12 reported a high level of weekly activity, and 2 reported a moderate level. Additionally, the expert wheelchair users presented a range of injury types, including T10 incomplete, T3 incomplete, C5–C7 incomplete, C7–C8 incomplete, and C7–C8 complete spinal cord injuries, neuromuscular autoimmune disorders, spina bifida, hemipelvectomy, cerebral palsy, multiple sclerosis, and weight-bearing bone breaks.

### 2.2. VO_2_ Tracking

**Figure 1:**
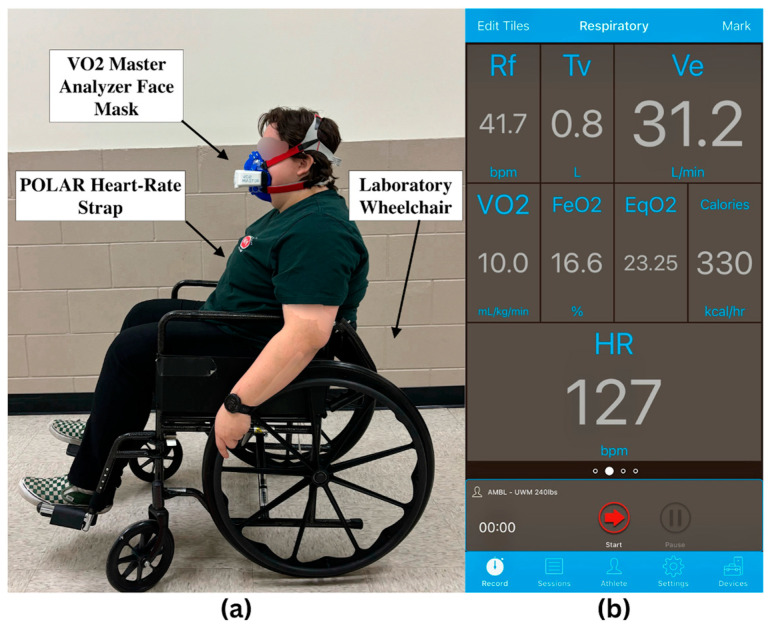
(**a**) Laboratory wheelchair used by a participant wearing a VO_2_ mask and heart rate monitor; (**b**) screenshot of the VO_2_ Master Manager phone application used to collect data from both sensors.

This study employed the VO_2_ Master Analyzer (VO_2_ Master Health Sensors Inc., Vernon, BC, Canada) to measure VO_2_ output. The device is portable and was attached to participants using a Hans Rudolph facemask and adjustable headgear, following the manufacturer’s instructions for use (Figure 1). Oxygen consumption is continuously monitored using electro-galvanic oxygen and differential flow sensors throughout the 6MPT. The VO_2_ data collected are used to assess and compare the participants’ aerobic exertion during the test.

### 2.3. Heart Rate Monitoring

A Polar H10 heart-rate strap (Polar Electro Oy, Kempele, Finland) was provided alongside the VO_2_ Master Analyzer to monitor heart rate (see Figure 1). The strap utilizes surface electrodes to detect the electrical signals from the heart during cardiovascular activity. The resulting electrocardiogram was then used to assess physiological exertion throughout the test.

### 2.4. Subjective Exertion Assessment

The modified Borg Perceived Rate of Exertion (RPE) scale was used at the end of the 6MPT, with participants being asked about their perceived physical effort during the test [30,31]. The RPE is subjective and based on physical cues one might observe during activities, like breathing, heart rate, perspiration, and muscle fatigue [30,31,32]. The answers range between 0 (rest) and 10 (maximal), with 1 representing very easy, 3 moderate, 5 hard, and 7 very hard.

### 2.5. Study Protocol

At the beginning of each lab appointment, participants received a complete explanation of the study protocol. After obtaining informed consent and completing the demographics questionnaire, the Polar HR strap was secured to each participant’s torso, horizontally below the sternum. The participant was then instructed to sit in an upright position, keep their eyes open, and refrain from talking while an observer recorded their resting heart rate for two minutes. Once completed, the VO_2_ mask was placed over the nose and mouth and secured to the participant’s head in preparation for the 6MPT. A lab wheelchair was provided for participants without a manual wheelchair of their own.

**Figure 2:**
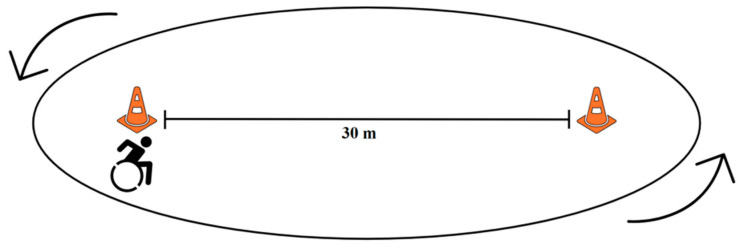
6-Minute Push Test Setup: 30 m long hallway marked with 2 cones. Participants began on one end and pushed themselves around the cones as many times as possible in 6 min.

The 6MPT was set up following a protocol outlined in our previous study [33]. The participant was directed to position themselves next to the first cone, facing the direction of the second cone 30 m down the hallway (Figure 2). The participant was then instructed to propel themselves as quickly as possible, turning around the second cone and returning to the starting cone. This process was repeated as many times as possible for the 6-minute duration. Throughout the test, the observer counted the number of complete laps and gave verbal cues at the beginning, middle, and end of the test.

At the completion of the 6MPT, the VO_2_ mask and Polar HR strap were removed from the participant. Then, the participant was asked to report their perceived level of effort during the 6MPT using the RPE scale. All participants received USD 25 as compensation for their participation and signed a reimbursement form upon completion of the study.

### 2.6. Data Processing

The VO_2_ and heart rate data were recorded and processed in the VO_2_ Master Manager application. All data were summarized into individualized reports. Each report consisted of the Max, Min and average of the participant’s heart rate and frequency of breath per minute, tidal volume, ventilation of oxygen, and fraction of expired oxygen recorded throughout the 6MPT.

### 2.7. Statistical Analysis

All statistical analyses were conducted using SPSS (version 29.0.1.0, SPSS Inc., Chicago, IL, USA). The Shapiro–Wilk test was performed to assess the normality of the variables: age, gender, resting heart rate, max heart rate, distance, RPE, and VO_2max_. As multiple variables significantly deviated from normality (*p* < 0.05), Spearman correlation analyses were used to assess relationships among key variables.

Group differences between expert and novice wheelchair users were examined using the Mann–Whitney U test, a non-parametric alternative to the independent samples *t*-test. To determine whether distance (a potential key predictor of VO_2max_) exhibited a significant interaction effect with group, a linear regression analysis with an interaction term was conducted. This analysis informed the decision to combine both groups for further regression analyses. Additionally, a chi-square test of independence was performed to determine whether gender distribution differed between groups. To identify the best predictors of VO_2max_, a stepwise linear regression was performed on the entire dataset, as well as on the expert group only to develop the most precise and practical model. Statistical significance was set at *p* < 0.05 for all tests.

## 3. Results

All recorded and reported variables in the 6MPT for 14 novice and 14 expert wheelchair-using participants are presented in Table 2.

**Figure 3:**
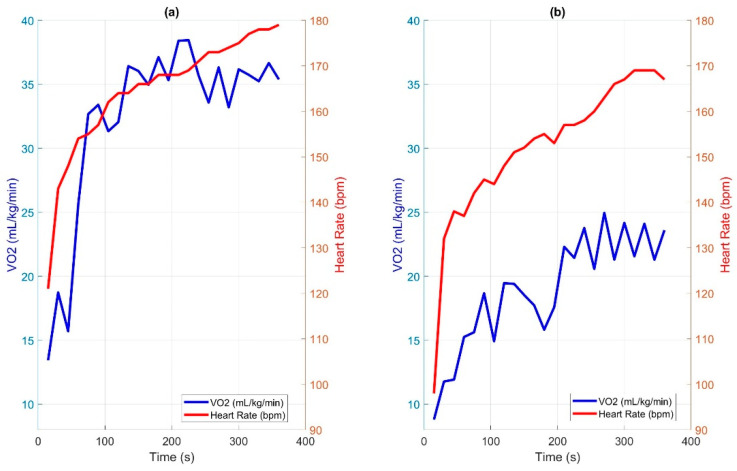
VO_2_ and heart rate data for two participants, both with a reported rate of perceived exertion (RPE) of 8: (**a**) expert, on the left; (**b**) novice, on the right, showing much lower VO_2_ values and slower increases in both heart rate and VO_2_, possibly due to a lower propulsion skill level.

The mean and standard deviation for each variable are presented for each of the groups and collectively for all 28 participants. Figure 3 exhibits the VO_2_ and HR data collected by the VO_2_ mask and heart rate monitor for an expert (Figure 3a) and a novice (Figure 3b) participant. Both participants reported an RPE of 8 at the end of the 6MPT.

### Statistical Analysis

The Shapiro–Wilk test indicated that multiple variables deviated from normality (*p* < 0.05); thus, non-parametric tests were used. The Mann–Whitney U test revealed that age (U = 49.5, *p* = 0.026) and distance traveled (U = 28.0, *p* < 0.001) were significantly different between the groups, with expert wheelchair users being older and covering more distance. No significant differences were found for resting heart rate, max heart rate, VO_2max_, or RPE (*p* > 0.05). The chi-square test of independence indicated no statistically significant difference in gender distribution between expert and novice wheelchair users (χ^2^(1) = 3.743, *p* = 0.053).

To further assess the relationship between distance and VO_2max_, an interaction effect between the groups was tested using a linear regression with an interaction term between group (novice vs. expert wheelchair users) and distance. The interaction term was found to be non-significant (β = 0.021, *p* = 0.336), allowing for the combination of both groups (N = 28) in subsequent regression analyses.

Spearman correlation analysis showed that distance (r = 0.685, *p* < 0.001) and RPE (r = 0.238, *p* = 0.045) were significantly correlated with VO_2max_, while max heart rate did not reach statistical significance (r = 0.205, *p* = 0.108).

Furthermore, a stepwise linear regression was used to explore the predictive relationships within the full sample. The following two models were identified for predicting VO_2max_:

- **Model 1:** Distance alone predicted **41.6%** of the variance (R^2^ = 0.416, adjusted R^2^ = 0.394, F(1,26) = 18.55, ***p* < 0.001**), with the following equation:

VO_2max_ = 7.164 + (0.024 × Distance) (1)

- **Model 2:** Including max heart rate improved the prediction to **56.1%** of the variance (R^2^ = 0.561, adjusted R^2^ = 0.526, F(2,25) = 16.00, ***p* < 0.001**), with the following equation:

VO_2max_ = −6.874 + (0.025 × Distance) + (0.091 × MaxHR) (2)

Both distance (*p* < 0.001) and max heart rate (*p* = 0.008) contributed significantly to Model 2, while RPE, despite its correlation with VO_2max_, did not significantly improve the model and was excluded.

Given that the stepwise regression analysis for the entire sample (N = 28) identified distance and maximum heart rate as significant predictors of VO_2max_, we further examined whether a model developed exclusively for the manual wheelchair expert group (N = 14) would provide a more targeted and practically useful estimation of their cardiorespiratory fitness. Since the primary application of this model is to assess wheelchair users’ health and performance, focusing solely on this group may improve its real-world relevance.

When considering only the wheelchair expert group, stepwise regression produced the following two models:

- **Model 1**: Distance alone predicted 55.0% of the variance (R^2^ = 0.550, adjusted R^2^ = 0.513, F(1,12) = 14.69, ***p* = 0.002**) with the following equation:

VO_2max_ = −0.649 + (0.033 × Distance) (3)

- **Model 2**: Adding max heart rate improved the model, predicting 70.9% of the variance (R^2^ = 0.709, adjusted R^2^ = 0.656, F(2,11) = 14.69, ***p* = 0.032** for the added value), with the following equation:

VO_2max_ = −12.781 + (0.028 × Distance) + (0.114 × MaxHR) (4)

## 4. Discussion

This study develops a predictive model to estimate VO_2max_ during the 6MPT for manual wheelchair users. The Spearman correlation analysis revealed that distance wheeled significantly correlated with VO_2max_ (r = 0.685, *p* < 0.001), reinforcing prior studies that 6MPT distance serves as a strong indicator of cardiovascular performance [17,18]. Additionally, the RPE showed a weaker yet significant correlation with VO_2max_ (r = 0.235, *p* = 0.045), whereas maximum heart rate did not reach statistical significance (r = 0.205, *p* = 0.108). These findings suggest that while both distance and RPE are associated with VO_2max_, heart rate may not be a sufficient standalone indicator of aerobic capacity for this test.

A stepwise linear regression identified two statistically significant models for VO_2max_ estimation from the 6MPT. Model 1, using only distance wheeled (F(1,26) = 18.55, R^2^ = 0.416, *p* < 0.001), provides a straightforward estimation method when heart rate monitoring is unavailable, while Model 2 offers a more comprehensive prediction (F(2,25) = 16.00, R^2^ = 0.561, *p* < 0.001) by incorporating an additional maximum heart rate variable.

To enhance the clinical relevance of the technique we also analyzed the predictive model using data from the expert wheelchair group alone, as this population is the most likely to benefit from its implementation in real-world practice. Despite the smaller sample size, the results were encouraging. Model 1, using only distance wheeled (F(1,12) = 14.69, R^2^ = 0.513, *p* = 0.002), provides a quick assessment of aerobic capacity when heart rate data were unavailable. Model 2, with added maximum heart rate further improved the model (F(2,1) = 14.69, R^2^ = 0.709, *p* = 0.032), making it particularly useful in settings where heart rate can be easily obtained, such as with wearable sensors. These findings strengthen the model’s utility by demonstrating its robustness within the target population. While the smaller sample size results in slightly lower statistical power, the model’s predictive value remains high, reinforcing its clinical applicability.

Given the relatively small sample size, we conducted a **post hoc power analysis** to determine whether the study had sufficient statistical power to detect the observed effects. The results indicate that both regression models for the entire sample (N = 28) and for expert wheelchair group alone (N = 14) had **high power (≥0.965)**, suggesting that the sample size was sufficient to detect meaningful relationships between predictors and VO_2max_.

Our regression models provide a practical and cost-effective alternative to expensive equipment that may not always be readily available, allowing clinicians and researchers to estimate aerobic capacity using distance alone (Model 1) or distance with heart rate (Model 2) from 6MPT. While VO_2max_ values derived from the 6MPT may not be accurate in an absolute sense, as that would require a separate VO_2_ test, they can serve as valuable trend indicators for monitoring cardiovascular health over time. This is particularly important for individuals with mobility impairments, for whom routine fitness assessments are often overlooked because of logistical challenges [21,26].

While the results are promising, the study does have some limitations. Notably, the expert group was significantly older and covered greater distances during the test compared to the novice group (*p* < 0.001). While age can be accounted for with a larger sample size, several factors could be contributing to the distance difference. First, some expert wheelchair users participated in their own personalized and/or sports wheelchairs that are designed for greater maneuverability, allowing for higher speeds and smoother turns, potentially contributing to increased distance covered. Second, higher propulsion skills due to everyday wheelchair use likely played a role in the greater distance covered during the test (Figure 3). Third, the expert group reported slightly higher weekly activity levels, which could further explain their cardiovascular endurance.

Additionally, the expert group had a higher proportion of males (3 females, 11 males) compared to the novice group (8 females, 6 males). This discrepancy reflects the observed trend of young wheelchair users (ages 18–64) having a higher proportion of males, as they are more prone to spinal cord and head injuries resulting in paralysis than females [34,35]. However, the chi-square test indicated that this difference in gender distribution was not statistically significant (χ^2^(1) = 3.743, *p* = 0.053), suggesting that gender alone may not fully explain the observed performance differences. Nevertheless, despite the significant group difference in distance traveled, the interaction term between group and distance was not significant (*p* = 0.336), indicating that the relationship between distance and VO_2max_ was comparable across groups. This allowed the data to be combined for model development. Although a post hoc power analysis indicates that our sample size was sufficient to detect the observed relationship, future studies should include a larger, more diverse cohort to improve the generalizability of the predictive model. External validation across different clinical and community settings will help ensure the models’ robustness.

While our model provides a cost-effective and accessible method for VO_2max_ estimation based on distance and heart rate, future research should explore other physiological parameters, for example, stroke efficiency, to further refine and improve the VO_2max_ estimation model.

## 5. Conclusions

This study developed a predictive model for VO_2max_ estimation in manual wheelchair users based on the 6MPT performance. The results show that distance wheeled during the test is a significant predictor of VO_2max_, with improved predictive power when maximum heart rate is included. The proposed models offer a practical, field-based alternative to traditional VO_2_ testing methods, which are often inaccessible for manual wheelchair users. By offering a simple and cost-effective approach to cardiovascular assessment, our model has the potential to improve routine long-term health monitoring of aerobic capacity in manual wheelchair users.

## Figures and Tables

**Table 1:** Demographics data for the two groups, novice (N = 14) and expert (N = 14) wheelchair users, and for the entire sample (N = 28). Total numbers are presented for gender, while the rest of the data are presented as the mean (standard deviation). BMI—Body Mass Index.

	Gender		Age (years)	Height (cm)	Weight (kg)	BMI
	Female	Male				
**Novice**	8	6	27 (8)	169 (8)	75 (13)	27 (5)
**Expert**	3	11	34 (13)	177 (12)	93 (27)	30 (9)
**Total**	11	17	31 (11)	173 (11)	84 (23)	28 (7)

**Table 2:** Data collected from the 6-Minute Push Test for the two groups of novice (N = 14) and expert (N = 14) wheelchair users. HR—heart rate; 6MPT—6 Minute Push Test; RPE—rate of perceived exertion. All collected parameters are reported with mean (standard deviation).

	Resting HR (bpm)	Max HR (bpm)	VO_2max_ (mL/kg/min)	Laps Completed	Total Distance (m)	RPE [1,2,3,4,5,6,7,8,9,10]
Novice	71 (12)	154 (30)	20 (6)	17 (4)	500 (113)	5 (2)
Expert	34 (13)	78 (17)	141 (33)	24 (9)	744 (204)	6 (2)
Total	75 (15)	148 (32)	22 (8)	23 (8)	622 (204)	6 (2)

## Data Availability

The original data presented in the study are openly available in FigShare at https://doi.org/10.6084/m9.figshare.28502258.v1 (accessed on 27 February 2025).

## Abbreviations

The following abbreviations are used in this manuscript:

6MPT	6-Minute Push Test
VO2max	Maximal Oxygen Consumption
HR	Heart Rate
RPE	Rated Perceived Exertion
BMI	Body Mass Index
SPSS	Statistical Package for the Social Sciences
IRB	Institutional Review Board
